# The effect of varying degrees of stenosis on transition to turbulence in oscillatory flows

**DOI:** 10.1007/s10237-022-01579-0

**Published:** 2022-04-20

**Authors:** Kartik Jain

**Affiliations:** Faculty of Engineering Technology, University of Twente, P.O. Box 217, 7500 AE Enschede, The Netherlands

**Keywords:** Lattice Boltzmann method, Transitional flow, Turbulence, Stenosis, Hydrodynamic instability

## Abstract

Many complications in physiology are associated with a deviation in flow in arteries due to a stenosis. The presence of stenosis may transition the flow to weak turbulence. The degree of stenosis as well as its configuration whether symmetric or non-symmetric to the parent artery influences whether the flow would stay laminar or transition to turbulence. Plenty of research efforts focus on investigating the role of varying degrees of stenosis in the onset of turbulence under steady and pulsatile flow conditions. None of the studies, however, have focused on investigating this under oscillatory flow conditions as flow reversal is a major occurrence in a number of physiologic flows, and is of particular relevance in cerebrospinal fluid flow research. Following up on the previous work in which a $75\%$ stenosis was studied, this contribution is a detailed investigation of the role of degrees of stenosis on transition in an oscillatory flow. A cylindrical pipe with $25\%$, $50\%$ and $60\%$ reductions in area in axisymmetric and eccentric configurations is studied for transition with 3 different pulsation frequencies of a purely oscillatory flow. Cycle averaged Reynolds numbers between 1800 and 2100 in steps of 100 are studied for each configuration resulting in 72 simulations each conducted on 76,800 CPU cores of a modern supercomputer. It is found that a higher degree of stenosis and eccentricity causes earlier transition to turbulence in oscillatory flow. The results further demonstrate that a higher frequency of oscillation results in larger hydrodynamic instability in the flow, which is more prominent in smaller degrees of stenosis.

**Supplementary Information:**

The online version contains supplementary material available at 10.1007/s10237-022-01579-0.

## Introduction

A stenosis refers to obstruction in a blood vessel or an anatomical segment like the subarachnoid space, spinal canal or respiratory airway. A stenosis can induce turbulence and flow separation in the blood vessel or the anatomical segment as a result of adverse pressure gradients. The presence of turbulence can eventually lead to secondary complications to the physiology in question. The onset of turbulence itself is likely to be influenced by the degree of stenosis itself as well as the nature of the flow, whether steady, pulsatile or oscillatory. For example, stenoses in the carotid arteries are well known risk factor for ischemic stroke (Fairhead and Rothwell 2005). The degree of stenosis has been established as a factor for stroke risk and indication for intervention (Qureshi et al. 2007). A recent review by Brinjikji et al. (2016) delineates the current advances in plaque imaging and association between degree of stenosis and the progression of plaque, while Berger and Jou (2000) reviewed studies of stenotic flows with a focus on atherosclerosis.

Previous studies have extensively investigated the complexity of fluid dynamics in stenosed vessels both numerically (Varghese et al. 2007a, b; Samuelsson et al. 2015) and experimentally (Ahmed and Giddens 1983, 1984). While studies of steady stenotic flow provide a general guidance about the nature of flow in such vessels, researchers try to assess the parts of cardiac cycles with most turbulent nature of the flow. In particular in the late systole when the flow decelerates, the flow has been shown to be highly disturbed, which stabilizes during the early diastole or acceleration phase of the cardiac cycle. The early studies of Yoganathan et al. (1986), Bluestein and Einav (1995) investigated in detail as to what degree of stenosis causes the flow to become turbulent.

Most of the studies of stenotic flows usually focus on uni-directional flows. This is natural because the areas where a stenosis are most relevant have a unidirectional steady or pulsatile flow. Earlier experiments of Ahmed and Giddens (1983) investigated post stenotic flow in a tube using LDA at mean Reynolds number of 600 and Womersley number ${\text{W}}_{{\text{o}}}   = 7.5$. They found strong turbulence and flow separation downstream of the $75\%$ stenosis. Ding et al. (2021) recently studied pulsatile flow in a 2D stenosed pipe using PIV experiments with a mean Reynolds number of 1750 and Womersley number of ${\text{W}}_{{\text{o}}} = 6.15$. They employed $25\%$, $50\%$ and $75\%$ degree of stenosis and found increasing turbulent intensities with increasing constrictions. Xu et al. (2017) carried out experiments in a sinusoidal shaped pulsatile pipe flow and observed that the critical Reynolds number increased with an increase in the Womersley number until ${\text{W}}_{{\text{o}}}= 2.5$. Then the critical Reynolds number reduced with a further increase in the Womersley number until it reached to ${\text{W}}_{{\text{o}}}=12$.

Some parts in the circulation however do experience a flow reversal. For example, the waveform of blood flow through arteries has been shown to change abruptly when passing from the thoracic aorta into the abdominal aorta in humans, exhibiting regions of flow reversal at the end of systole. Holenstein and Ku (1988) developed a lumped parameter model of such a flow and referred to it as *triphasic* flow with positive-negative-positive parts of the cardiac cycle. Another form of such a triphasic flow is a purely oscillatory flow, known to occur mainly in the central nervous system (CNS) namely the circulation of the cerebrospinal fluid (CSF). Another purely oscillatory flow is the airflow in the respiration system. In a purely oscillatory flow the underlying factors that trigger turbulence, the distribution of turbulence in various parts of the cycle, as well as the stabilization mechanisms are different than those from a steady or pulsatile flow with no reversal component. Earlier experiments by Hino et al. (1976, 1983) have investigated detailed characteristics of turbulence in oscillatory pipe flow. Other similar studies (Sarpkaya 1966; Yellin 1966; Iguchi and Ohmi 1982) addressed such aspects but also in simple pipes without any distortion that would resemble an anatomical geometry.

In a recent work Jain (2020b) we investigated transition to turbulence in a purely oscillatory flow with three pulsation frequencies in a pipe with $75\%$ area reduction in eccentric and axisymmetric configurations. While the role of symmetry of the geometry and the pulsation frequencies in the onset of turbulence was the focus of that study, the degree of stenosis itself was not investigated. The present contribution investigates the characteristics of turbulence in a stenosed pipe with area reductions of $60\%$, $50\%$ and $25\%$. All three degrees of stenosed pipes are studied in two configurations: one that is symmetric to the principal axis of the pipe while the other which is offset by 0.05 diameters of the pipe to introduce an eccentricity or a geometric aberration. Three different flow oscillation frequencies with Womersley numbers ranging between $\sim$ 5 and 8 are studied in all the degree and configurations of the stenosis to explore detailed characteristics of flow transition. Reynolds numbers between 1800 and 2100 are investigated in steps of 100 to compare the characteristics of flow transition with varying degrees of stenosis against the previous study (Jain 2020b).

The three main focal points of the study namely *degree of stenosis*, *pulsation frequencies* and *symmetry/asymmetry of the geometry* are representative of a broad range of CSF and respiratory flows. For example stenosis of the lumbar spine ranges from various degrees within the range studied here (Genevay and Atlas 2010) while the obstruction to CSF caused by Chiari I malformation also lies in this range (Linninger et al. 2016). Subglottic stenosis is a narrowing of a specific portion of the windpipe (trachea) known as the subglottis (just below the vocal cords). These are classified as grade 1 stenosis with a luminal obstruction $<50\%$, grade 2 and 3 stenosis with 51–70% and 71–99% obstruction respectively (Jefferson et al. 2016). The pulsation frequencies range from a Womersley number of approximately 5 (CSF) to 11 (respiratory airflow), which corresponds to the pulsation frequencies studied in this work. Occurrence of stenosis in a symmetric fashion is rare due to the complexity of the anatomy and most obstructions are found in a highly asymmetric fashion. Similarly, the Reynolds numbers, mainly due to the presence of stenosis can reach up to 2000 in CSF and 1500–8000 in respiratory airways. The present study is thus a generalization of stenotic flow in these two applications. The idea is to introduce representative anatomic and physiologic complexity while maintaining simplicity that could allow prospects for future studies in identifying the chances of turbulence. The goal for these canonical studies is to serve as benchmark for future comparisions and thus a patient specific anatomic case has not been studied.

For the absence of a clear definition for a flow regime that is neither laminar nor turbulent, we have addressed the flow in this study as transitional. Thus, if fluctuations occur in localized parts of the domain during parts of the cycle, we term the flow transitional. This is in accordance with several such studies in this direction (Varghese et al. 2007b; Samuelsson et al. 2015; Yellin 1966; Jain 2020a, b). The onset of a fully developed turbulence is not pursued and the focus is on exploring the critical Re at which the flow leaves a laminar regime for a particular degree of stenosis, and a specific pulsation frequency (Womersley number). The Reynolds number at which the flow transitions is judged on the basis of hydrodynamic instabilities in the velocity field and deflection of the jet downstream of the stenosis.

We have employed the lattice Boltzmann method (LBM) for the DNS of this study as the method scales well on parallel computers—allowing for accurate capture of transitional flow characteristics. The LBM when setup properly (Jain 2020a) is a very appropriate second order accurate method for the DNS of flow. While the order of accuracy is lower than competitive higher order methods, the strict control of numerical viscosity in LBM allows for a relatively easier capture of flow fluctuations. A comparison of LBM with other second-order accurate methods (Marié et al. 2009) has found that the numerical dissipation in LBM, even at the scales of grid spacing and the numerical dispersive effects are relatively smaller.

## Methods

**Fig. 1:**
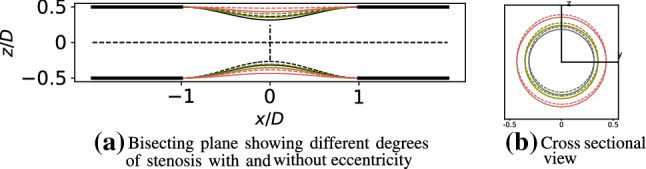
Left: A bisecting plane showing the principal pipe with varying degrees of stenoses where gray, green and red respectively represent $60\%$, $50\%$ and $25\%$ reduction by area. Corresponding dotted lines show the offset of 0.05 diameters of the principal pipe to introduce an eccentricity in each case. Right: Cross sectional view of different degrees of stenoses

The geometry of the stenosis was adopted from the experiments of Ahmed and Giddens (1984) (shown in Fig. 1). Stenosis of varying degrees in axisymmetric and eccentric configurations were created using the Blender software previously by Gericke (2016). The gray, green and red lines respectively demonstrate $60\%$, $50\%$ and $25\%$ reduction in area whereas there dotted counterparts indicate the eccentricity that was introduced.

Similar to previous study Jain (2020b) the pre and post-stenotic regions of the vessel were *equally* extended by 12 vessel diameters to account for the reversing nature of the flow, which resulted in a total pipe length of approximately 26 diameters. Due to flow reversal, the fluid travels equally on both sides of the stenosis throat, and the location of stenosis in the pipe is thus expected to influence the onset of transition as well as propagation of formed turbulent bursts.

### Oscillatory flow

The oscillatory nature of the flow can be described by the Womersley solution with a negative (flow reversal) component. The Womersley solution for laminar, pulsatile flow through rigid tubes is defined as:

1 $$ \left. {\begin{array}{*{20}l}    {\frac{{u_{x} }}{{u_{{\text{c}}} }} = A\left[ {1 - \frac{{J_{0} (i^{{3/2}} \alpha 2r/D)}}{{J_{0} (i^{{3/2}} \alpha )}}} \right]\sin (\omega t),} \hfill  \\    {\frac{{u_{y} }}{{u_{{\text{c}}} }} = 0} \hfill  \\    {\frac{{u_{z} }}{{u_{{\text{c}}} }} = 0} \hfill  \\   \end{array} } \right\} $$

where $u_\mathrm{c}$ is the half-cycle-averaged inlet centerline velocity,* A* and $\omega$ respectively are the amplitude and angular frequency of pulsation, $J_0$ is the Bessel function of first kind and zeroth order, and $\alpha$ is the non-dimensional Womersley parameter ($= \frac{1}{2}D \sqrt{\omega /\nu }$, where $\nu$ is the kinematic viscosity). The Womersley parameter defines the extent to which the laminar profile departs from quasi-steadiness. This effect becomes significant when the Womersley parameter, $\alpha = 3$. Three different frequencies of pulsation were studied where $\omega _2=2 \omega _1$ and $\omega _3=4 \omega _1$. The forward and backward flow are referred to as *blowing* and *suction* stages respectively in the following.

**Fig. 2:**
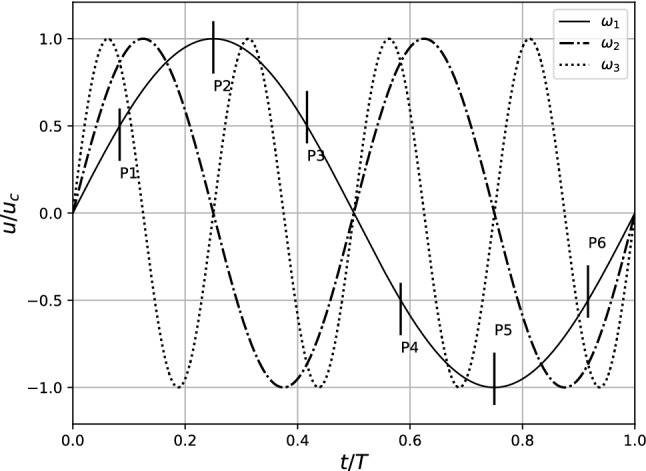
Axial centerline velocity at the vessel inlet with three different oscillation frequencies, $\omega _1$, $\omega _2$ and $\omega _1$. Observations were made during 6 equidistant points along the sinusoidal cycle (shown only for $\omega _1$)

Figure 2 depicts the flow waveform and the 6 time intervals of* T*/6 where flow quantities were analyzed. Like the studies of Jain (2020b), the flow profile here consists of a negative component which accounts for the flow reversal, and the mean flow in this case is thus zero describing a purely oscillatory flow. The Reynolds number was based on the main vessel diameter, *D*, and the mean inlet centerline velocity of *half the cycle*, $u_\mathrm{c}$. The Re was varied from 600 to 2100 in steps of $\delta {\text{Re}} = 100$ for both the configurations of the stenosis and three pulsation frequencies. This resulted in a total 90 sets of simulations.

The value of A and $\alpha$ in Eq. 1 were 0.667 and 5.4 respectively. For the lowest pulsation frequency $\omega _1$ the Womersley parameter $\alpha _1$ was chosen as 5.4 keeping the frequency of oscillations of the cerebrospinal fluid (CSF) in view. To attain the pulsation frequencies of $\omega _2=2 \omega _1$ and $\omega _3=4 \omega _1$, the corresponding Womersley parameters became $\alpha _2=7.636$ and $\alpha _3=10.8$ respectively. Zero pressure and velocity were set at every lattice cell as initial conditions. A zero pressure gradient was maintained at the outlet of the pipe which translates to an extrapolation of incoming populations at the outlet (Junk and Yang 2011). The flow waveform shown in Fig. 2 was prescribed at the inlet. Flow was allowed to develop for two initial cycles before analysis was performed.

### Direct numerical simulation

The adaptable poly engineering simulator (APES) framework (Roller et al. 2012; Klimach et al. 2014; Qi et al. 2016) was chosen for the simulations which contains a full tool-chain of simulation software ranging from the mesh generator *Seeder* (Harlacher et al. 2012) and the LBM solver *Musubi* (Hasert et al. 2014). Modules within APES are available as an open source software tool for academic research.^1^ The *Musubi* LBM solver is mainly managed at the Institute of Software Methods for Product Virtualization within the German Aerospace Center (DLR) in Dresden, Germany. Extensive development and use of the solver for applications in physiology is done at the University of Twente, The Netherlands.

Extensive verification of the LBM solver *Musubi* has been performed in prevous works (Hasert et al. 2014). About 30 verification test cases are executed automatically every night that ensure that the physics computation and parallel as well as serial performance of the solver compares well with the benchmarks. Several efforts in the direction of validation have been placed, see for example a comparison against experiments reported in Johannink et al. (2015). A comprehensive validation for the simulation of physiologic flows in transitional and turbulent regime was conducted using the benchmark set out by the US Food and Drug Administration (FDA) (Jain 2020a). For each new biomedical application, comparisons against in vitro and in vivo measurements are performed during the continuous use and development of *Musubi*. See for example, a recent work conducted in parallel in which simulation results were compared against *in vitro* experiments of nasal airflows (Hebbinket al. 2022).

Due to the simplicity of the geometry studied in this work, the employment of the Bhatnagar–Gross–Krook (BGK) relaxation scheme was sufficient. The BGK relaxation parameter was set to $\Omega = 1.84$ in the present study that keeps the lattice Mach number within the stability limits of the LBM (Junk et al. 2005). Further details on these aspects can be referred elsewhere (Jain 2020a).

The spatial and temporal resolutions were chosen as $\delta x = 32 \times 10^{-4}$ and $\delta t = 7.5 \times 10^{-6}$ units respectively. Based in the degree of stenosis, this resulted in $\sim$ 700 million cells. When the pipe had the minimum degree of stenosis ($25\%$), there was more volume of the fluid resulting in approximately 1 million more lattice cells than the most severe case. Presuming the main vessel diameter, *D*, of 1 unit, the spatial resolution $\delta x$ resulted in 124, 156 and 232 cells along the diameters of the throat for $60\%$, $50\%$ and $25\%$ degree stenoses respectively. The number of cells along the main diameter of the parent pipe was 312. This ensured the symmetry of the mesh. It may be remarked that the number of lattice cells here does not translate directly to the *order* of polynomial or *resolution in space* for other higher order methods like the spectral methods. We employed the D3Q19 stencil of the LBM in this study, which means that each lattice cell had 19 degrees of freedom. In higher order methods, the order of the polynomial allows for a higher accuracy within a cell, which is not the case with LBM. Thus these resolutions cannot be compared with those employed in similar studies (Varghese et al. 2007b). A detailed comparison of methods is left for future efforts.

Our previous works Klimach et al. (2014), Hasert et al. (2014), Qi et al. (2016) have shown that the *Musubi* LBM solver exhibits excellent weak and strong scaling as well as parallel efficiency of more than $90\%$ on supercomputers when every core has more than about 4000 lattice nodes. Simulations were thus executed on 76,800 cores of the *SuperMUC-NG* supercomputer installed at the Leibniz Supercomputing Center in Munich, Germany. With this setup each core (or MPI rank) had about 9000 lattice nodes for computations thereby reducing the communication to computation ratio to an optimal minimum, and ensuring fully efficient utilization of compute resources.

Simulations at Reynolds of 2000 and 2100 are only reported in this work due to the laminar nature of the flow at lower Reynolds numbers. The simulations for all the cases were first conducted for only $n=4$ cycles as in case of laminar flow, computation of more cycles was redundant. Some of these simulations were conducted using 7200 CPUs of the Snellius system, which is the national supercomputer in The Netherlands. In case of a transitional or a turbulent flow, it is always challenging to decide how many total cycles should be used for ensemble averaging until the quantities are statistically converged as the compute costs and physical details need to be balanced. Ideally, the ensemble average should attain the shape of a laminar flow, similar to inflow or the turbulent fluctuations should wash out from the averaged plot.

Due to the lack of this information a priori, we first simulated the flow with pulsation frequency $\omega _1$. In the cases where flow transition was identified, we restarted those simulations from the 4th cycle onwards and allowed the computation of further $n=20$ cycles resulting in a total of $n=24$ cycles. For higher pulsation frequencies, due to the same time step, the number of cycles automatically became $n=48$ for $\omega _2$ and $n=96$ for $\omega _3$. Each cycle required $\sim$ 4.3 min of execution time. An *abort criteria* was set in *Musubi* to stop the simulation upon achievement of a steady ensemble average. Thus, each subsequent cycle was ensemble averaged with the previously computed cycles and when the velocity fluctuations in ensemble averages vanished, the simulations were stopped automatically. This analysis revealed that a total of about $n=20$ cycles were enough for the most turbulent simulation, which further implicated the choice of $n=20$ cycles for all the cases. Based on this a total of $n=22$ cycles were computed for each simulation and the last 20 were used for the analysis of turbulent flow characteristics. It may be noted that 20 cycles are relatively less than what would be needed for statistical convergence. A recent study by Andersson and Karlsson (2021) reports that employment of much larger number of cycles is required for statistical convergence. The 20 cycles in this case are sufficient because of the zero mean nature of the flow, which is absent in a pulsatile blood flow reported in the study of Andersson and Karlsson (2021).

### Flow characterization

A total of $n=22$ (where initial 2 cycles are discarded from analysis) cycles were computed for each Re simulation. Velocity over these cycles was ensemble averaged for the analysis of turbulent characteristics as:

2 $$\begin{aligned} {\overline{u}}(x,t) = \frac{1}{n}\sum _{k=0}^{n-1} u (x,t + kT) \end{aligned}$$

where *u*(*x*, *t*) is the instantaneous velocity at a particular lattice site, **x** denotes the spatial coordinates, **t** is the time and **T** is the period of cycle.

We assumed that turbulence was the only source of fluctuations in the flow and composed the velocity field into a mean and a fluctuating component. This is known as Reynolds’ decomposition and is commonly used for steady flows. The oscillatory flows have a reversal component in addition to the pulsating nature of the flow. Triple decomposition (Hussain and Reynolds 1970) is thus considered a more suitable method for the analysis of such flows:

3 $$\begin{aligned} u_i(x,t) = \bar{u_i}(x) + u_{i}^{\prime }(x,t) + \tilde{u_{i}}(x,\phi ) \end{aligned}$$

Thus, a periodic component denoted by $\tilde{u_{i}}(x,\phi )$ gets added to Reynolds’ decomposition, which is a function of the time within the cycle. The phase is defined as $\phi = mod(t/T,1)$ where* T* is the total length of the cycle. Cycle-to-cycle variations are also intrinsically taken into account by the method of triple decomposition.

The Turbulent Kinetic Energy (TKE) is derived from the fluctuating components of velocity in 3 directions as:

4 $$\begin{aligned} k = \frac{1}{2}\Big ( {u_{x}^{\prime 2} + u_{y}^{\prime 2} + u_{z}^{\prime 2}} \Big ) \end{aligned}$$

The frequency components present in a transitional or turbulent flow can be analyzed from the power spectral density (PSD) of the TKE. The PSD was computed in this work using the Welch’s periodogram method, which was related with Kolmogorov’s energy decay to observe the inertial and viscous ranges.

### Kolmogorov microscales

In the previous Jain (2020b) as well as in related work Helgeland et al. (2014) the Kolmogorov theory has been taken as a reference to estimate the quality of the mesh employed for DNS. It may be noted that the degrees of stenosis considered in this article are relatively lower than those in the previous work where stenosis with a $75\%$ area reduction was chosen. This implies that the amount of turbulence (if any) should be lower in the present work, which in turn suggests that the resolution employed in simulations should be enough for the accurate capture of transitional characteristics. It was however not a priori known whether turbulence will occur in these cases at Reynolds of up to 2100 or not. This means that simulations at even higher Reynolds number could have been required and thus Kolmogorov microscales were still calculated,

The Kolmogorov scales are defined as spatial and temporal scales when viscosity dominates and the TKE is dissipated into heat (Pope 2000).

The Kolmogorov scales, non-dimensionalized with respect to the velocity scale $u_\mathrm{c}$ and the length scale *D* are computed from the *fluctuating* component of the non-dimensional strain rate defined as:

5 $$\begin{aligned} s^{\prime }_{ij} = \frac{1}{2}\bigg (\frac{\partial u_{i}^{\prime }}{\partial x_j} + \frac{\partial u_{j}^{\prime }}{\partial x_i}\bigg ) \end{aligned}$$

The Kolmogorov length, time and velocity scales are then respectively computed as:

6 $$\begin{aligned} \eta&= \bigg ( \frac{1}{\hbox {Re}^2} \frac{1}{2 s^{\prime }_{ij}s^{\prime }_{ij}} \bigg )^{1/4} \end{aligned}$$

7 $$\begin{aligned} \tau _{\eta }&= \bigg ( \frac{1}{2 s^{\prime }_{ij}s^{\prime }_{ij}}\bigg )^{1/2} \end{aligned}$$

8 $$\begin{aligned} u_{\eta }&= \bigg ( \frac{2s^{\prime }_{ij}s^{\prime }_{ij}}{\hbox {Re}^2} \bigg )^{1/4} \end{aligned}$$

Based on these scales, the quality of the spatial and temporal resolution of a simulation is estimated by computing the ratio of $\delta x$ and $\delta t$ against the corresponding Kolmogorov scales i.e.

9 $$\begin{aligned} l^{+} = \frac{\delta x}{\eta } \quad t^{+} = \frac{\delta t}{\tau _{\eta }} \end{aligned}$$

The $l^{+}$ and $t^{+}$ were computed using Eq. 9 for only the $60\%$ stenosis case at the highest frequency as the fluctuations were highest in this case. The values of these scales were 1.18 and 0.62 respectively.^2^

## Results

**Fig. 3:**
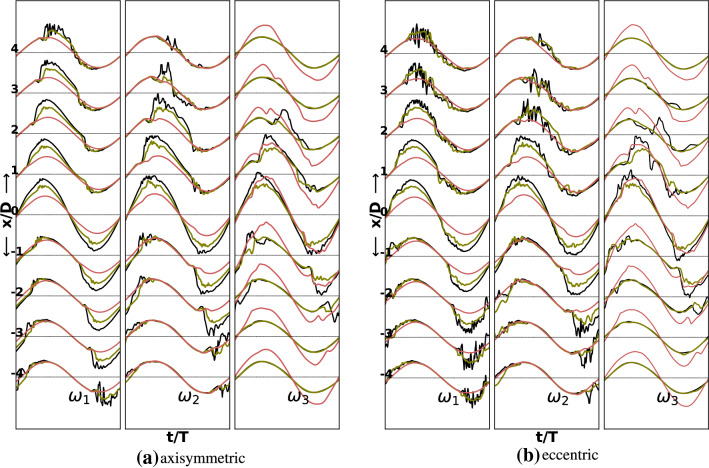
Normalized axial centerline instantaneous velocity during the last cycle for three degrees of stenoses and three oscillation frequencies in the axisymmetric and the eccentric cases at Re $=$ 2000. For oscillation frequencies, $\omega _1$ and $\omega _2$ last two and four cycles are shown. The black, green and red lines respectively correspond to $60\%$, $50\%$ and $25\%$ stenosis degrees

**Fig. 4:**
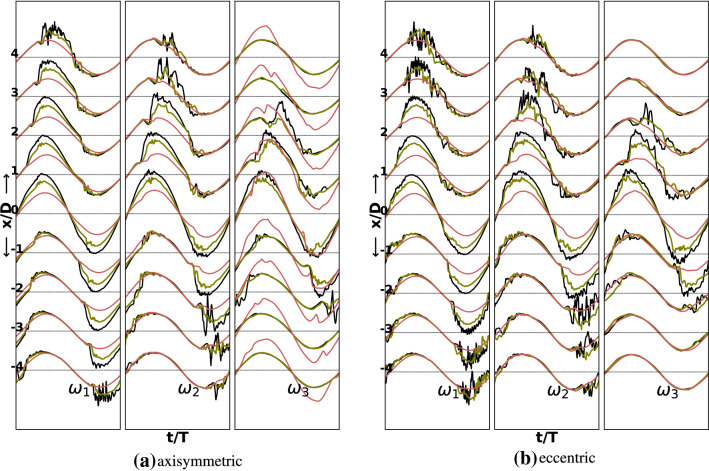
Normalized axial centerline instantaneous velocity during the last cycle for three degrees of stenoses and three oscillation frequencies in the axisymmetric and the eccentric cases at Re $=$ 2100. For oscillation frequencies, $\omega _1$ and $\omega _2$ last two and four cycles are shown. The black, green and red lines respectively correspond to $60\%$, $50\%$ and $25\%$ stenosis degrees

The flow at $\hbox {Re}=1800$ was laminar and it started to show valleys and peaks in the centerline velocities at $\hbox {Re}=1900$ while at $\hbox {Re}=2000$ these valleys and peaks became more pronounced and minor fluctuations started to appear mostly in the $60\%$ case. The TKE components up to this Reynolds number were depictive of a laminar flow with minor vortex shedding in the $60\%$ eccentric stenosis case. The flow only transitioned to weak turbulence at $\hbox {Re}=2100$ commensurate with the previous study (Jain 2020b) of $75\%$ stenosis. Figures 3 and 4 shows traces of instantaneous centerline velocity along the streamwise direction for axisymmetric and eccentric cases at $\hbox {Re}=2000$ and $\hbox {Re}=2100$ respectively.

Clearly the fluctuations at $\hbox {Re}=2100$ are markedly higher than those at $\hbox {Re}=2000$ for the $60\%$ and $50\%$ degrees of stenosis. The flow in both stenosis configurations is essentially laminar for the $25\%$ stenosis. At a higher oscillation frequency of $\omega _3$, minor distortions in the sinusoidal profile however start to appear even in the $25\%$ case. For the $50\%$ stenosis case the flow shows minor fluctuations during the peak flow and the deceleration phase. In the eccentric case these fluctuations are relatively higher at this degree of stenosis. The flow attains a transitional character only at a stenosis degree of $60\%$ for both the stenosis configurations. The fluctuations are relatively higher in the eccentric case. The regions downstream of the stenosis where the flow transitions and thereafter stabilizes appears to shift closer to the stenosis throat with increasing oscillation frequency. For the eccentric stenosis, the regions of maximum chaos are mostly confined in the locations $2D<x<4D$, $1D<x<3D$, $0D<x<2D$ respectively for oscillation frequencies $\omega _1$, $\omega _2$ and $\omega _3$. In the axisymmetric case, however, the regions of jet breakdown are shifted further downstream by one diameter whereas the regions of restabilization are the same as in eccentric stenosis for all the studied frequencies. At the highest studied frequency, $\omega _3$, there are signs of fluctuations even at the stenosis throat ($x=0D$), which are a result of fast oscillation of the fluid that transfers the vortices on both sides of the stenosis rapidly.

**Fig. 5:**
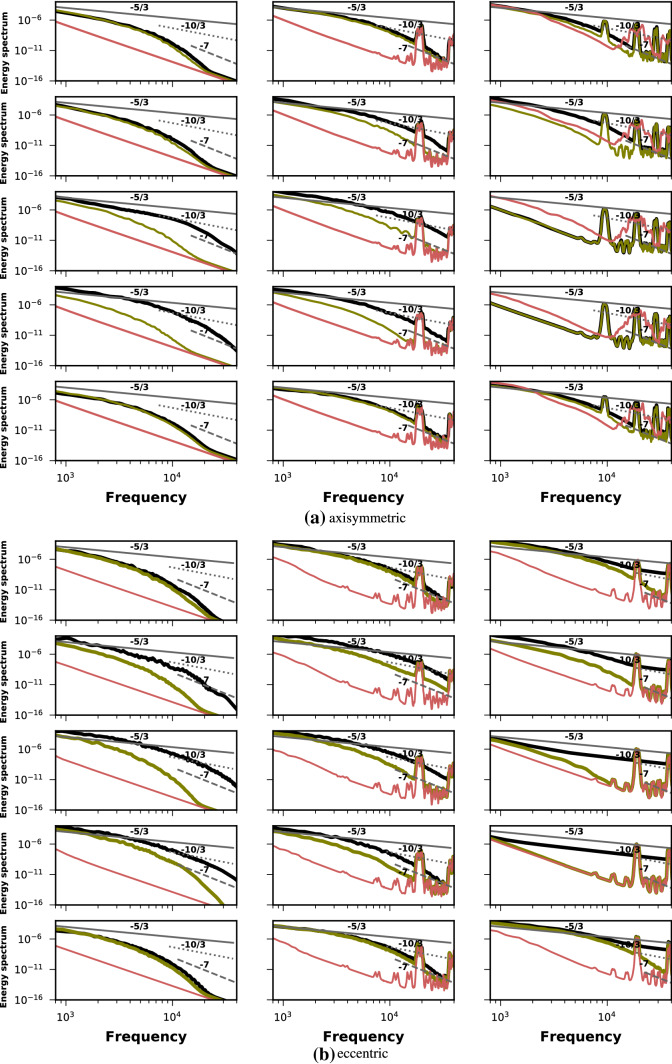
Power spectral density of the turbulent kinetic energy in axisymmetric and eccentric cases at $\hbox {Re}=2100$. The black, green and red lines respectively correspond to $60\%$, $50\%$ and $25\%$ stenosis degrees. Each column shows the PSD at $x=1,2,3$ and 4 diameters downstream of stenosis respectively during the blowing stage. Each rows from left to right shows PSD for oscillation frequencies of $\omega _1$, $\omega _2$ and $\omega _3$ respectively. The solid, dotted and dashed lines have slope of $\frac{-5}{3}$, $\frac{-10}{3}$ and $-7$

These findings are further elaborated in the power spectral density (PSD) plots of Fig. 5. Each column from top to bottom shows the PSD of the TKE at the stenosis throat and 4 locations downstream of it during the blowing stage whereas the black, green and red lines respectively demonstrate the PSD for $60\%$, $50\%$ and $25\%$ degrees of stenoses. In the axisymmetric case for oscillation frequency $\omega _1$, the spectrum at $x=2D$ and $x=3D$ begins to take a shape with few frequencies in the inertial range depicted by the $-5/3$ decay. At frequencies higher than $10^3$ the spectrum rolls off to $-10/3$ and subsequently to $-7$. The spectrum at $x=4D$ is similar for $60\%$ and $50\%$ degree of stenosis while the flow in $25\%$ case is laminar. Large valleys and peaks (red line) are seen in $50\%$ and $25\%$ stenoses at higher oscillation frequencies of $\omega _1$ and $\omega _2$ depictive of the minor disturbances that were seen in instantaneous traces as well. Similar observations can be drawn from the PSD plots of the eccentric case with a clear indication of larger turbulent activity. There are larger number of frequencies in the $-5/3$ range and the roll of to $-10/3$ and $-7$ happens later in case of eccentric stenosis.

### Vortex structures

**Fig. 6:**
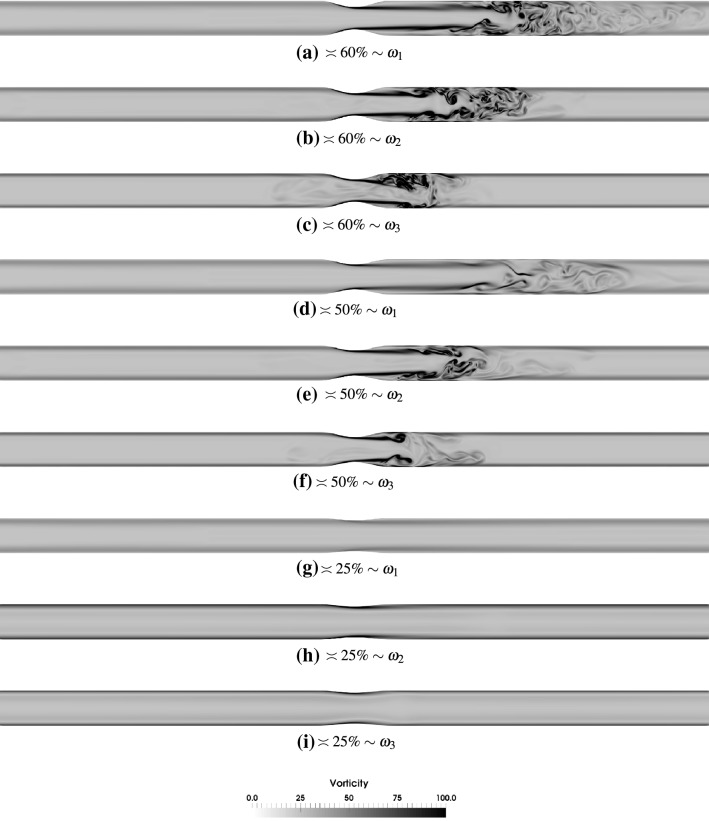
Instantaneous vorticity magnitude at the observation point P3 across bisecting planes in the axisymmetric stenoses of various degrees for $\hbox {Re}=2100$ and oscillation frequencies $\omega _1$, $\omega _2$ and $\omega _3$. The vorticity is normalized by $u_{{\text{c}}} /D$

**Fig. 7:**
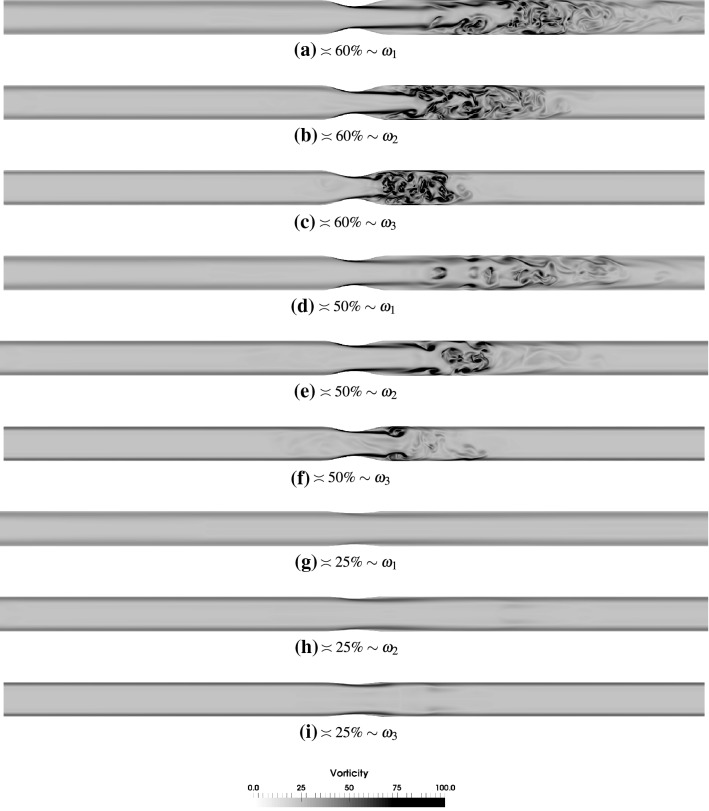
Instantaneous vorticity magnitude at the observation point P3 across *x*–*z* bisecting planes in the eccentric stenoses of various degrees for $\hbox {Re}=2100$ and oscillation frequencies $\omega _1$, $\omega _2$ and $\omega _3$. The vorticity is normalized by $u_c/D$

Figures 6 and 7 show the profiles of vorticity magnitude across a bisecting plane in the axisymmetric and the *x*–*z* bisecting plane in the eccentric stenoses cases of all variants and oscillation frequencies. The observation point P3 (see Fig. 2) has been chosen as the flow exhibits maximum chaos at this observation point in the sinusoidally oscillating flow. The suction stages (points P4–P6) have been omitted because more or less similar profiles are seen on the left side of the stenosis throat.^3^ The previous observation that the regions of jet breakdown and restabilization shift closer to the stenosis throat with increasing oscillation frequencies are clearly seen in the vorticity plots.

In the axisymmetric case the jet that emanates from the throat has enough time to travel downstream at oscillation frequency $\omega _1$ before its breakdown and transition of flow to weak turbulence. At higher frequencies, the flow breaks down closer to the throat as the deceleration phase commences sooner in these cases. It may be specifically seen that at $60\%$ stenosis and oscillation frequency $\omega _3$ (Fig. 6c) the jet is deflected towards the lower part downstream of the stenosis in this particular cycle. This is a consequence of the fact that when the flow reverses its direction, the vortices merge just downstream of the throat. The frequency ($\omega _3$) is relatively high that breaks the symmetry that is otherwise present at lower frequencies. The momentum of the flow is suddenly lost when it reverses its direction and this happens at a rapid speed at this frequency resulting in this loss of symmetry even in the axisymmetric case. This is also seen to a certain extent in $50\%$ stenosis with $\omega _3$ (Fig. 6f) albeit at a lower extent due to the lower intensity of turbulence in the first place due to less constriction in the pipe. For the $25\%$ stenosis case this phenomenon is never observed as the flow is clearly laminar for all the oscillation frequencies.

The eccentricity of the stenosis causes the jet to deflect earlier than the axisymmetric case resulting in a jet breakdown location closer to the stenosis throat. Here a higher oscillation frequency amplifies the chaos and factors that influence the onset of transition, in addition to eccentricity, are faster blowing and suction as was stated above. For the $50\%$ stenosis case at oscillation frequency $\omega _1$ (Fig. 7f), the jet does not appear to be deflected as a result of eccentricity but an inflexion point is created about 2.5 diameters downstream of stenosis where the turbulent activity occurs and extends beyond to up to about 4 diameters. At higher frequencies in this degree of stenosis, interesting patterns are observed with a similar fluid dynamical behavior as in axisymmetric stenosis.

## Discussion

The direct numerical simulations of oscillatory flow in varying degrees of stenosis in axisymmetric and eccentric configurations have found a correlation between severity of the stenosis and the intensity of turbulence in an oscillatory flow. This influence is a well established fact but none of the previous studies have characterized this for oscillatory flow.

In comparison to the previous study with $75\%$ stenosis (Jain 2020b), this study has contrastingly found that the increasing frequencies of oscillation result in earlier breakdown of the flow, a phenomenon most pronounced in lower degrees of stenosis. In a constriction as large as $75\%$ the onset of turbulence was predominated by the geometry rather than the complexity of the flow field. Upon reducing the constriction in the present study the frequencies of the oscillatory flow field resulted in the onset and sustainment of turbulent flow. At high frequencies of oscillation, the instant at which the downstream flow reversed direction had a lag in different radial directions resulting in merger of vortices and creation of newer ones.

The axisymmetric or the eccentric nature of the geometry here also had less pronounced effect in comparison to the previous study, mainly because of the aforementioned factors. The most interesting distinction between eccentric and axisymmetric cases is seen in $25\%$ degree of stenosis and pulsation frequency $\omega _3$. As can be seen from Fig. 7h, i miniature patches of vortices are created downstream of the stenosis. These vortices that are shed are a consequence of jet deflection, which sows the seeds of flow transition already at this small constriction in the pipe. These seeds are not intense enough to cause a breakdown of the flow at this Reynolds number, and the flow reversal further adds to their incapability in bringing about flow transition.

The intermittent nature of turbulence along the flow cycle demonstrates how flow reversal stabilizes the flow field—commensurate with the previous study (Jain 2020b). The centerline velocities however demonstrate different characteristics with different oscillation frequencies. At larger pulsation frequencies $\omega _2$ and $\omega _3$ the turbulence has more a decaying nature because the deceleration phase has a reduced time with increasing frequencies.

One of the most surprising observations stems from a comparison of Figs. 6c and 7c. While the former figure is for axisymmetric case and the latter for eccentric case, the shape of the flow jet seems to suggest as if these figures have been interchanged. The time lapse between events like flow jet breakdown, reversal and stabilization is extremely small in these cases due to the high frequency of the sinusoidal waveform. Due to that the vortices that were created during the deceleration phase are immediately disturbed by the flow reversal that tries to stabilize the flow field and push the flow in the other direction. A fast merger and annihilation of vortices breaks the shape of the post stenotic jet giving rise to this behavior. If we take a closer look at Fig. 7c we can observe that the jet is actually deflected upwards due to the eccentricity.^4^

From the PSD plots (Fig. 5) it may be seen that whatever turbulent nature of the flow there is, it is less intense than that for $75\%$ stenosis. At higher degrees of stenoses the PSD attains a $\frac{-5}{3}$ decay albeit for a very small range of frequencies. A roll of to $\frac{-10}{3}$ and $-7$ happens much sooner demonstrating the decaying nature of turbulence.

The degree of constriction, in almost all pathologies indicates the severity of the situation. Bluestein and Einav (1995) focused their study with background of heart valves and for pulsating flow their general observation, that a higher constriction results in larger turbulence, was consistent with the results of the present study. It may be queried how relevant a zero mean oscillatory flow is in physiology. Holenstein and Ku (1988) has demonstrated the relevance of reverse flow in infrarenal vessels as well albeit the flow there only has a reversed component and the mean of the flow is not zero. This study will thus have a main relevance in CSF and respiratory flow research that are purely oscillatory.

This study has several limitations, most of which are similar to the previous study (Jain 2020b). In the context of degrees of stenosis it may be mentioned that despite the study of three different pulsation frequencies, the waveform has always been sinusoidal. In both CSF and respiratory flows, the waveforms are very complex due to a number of physiologic factors. Such waveforms might result in alternate turbulence mechanisms. Also, despite eccentricity the stenosis shape is still symmetric, which is unlikely to happen in an anatomic case. This study may thus be inferred as one where complexity of the anatomy has been incorporated in the form of major factors that are present, while maintaining the constitutive simplicity so that these results can act as a benchmark.

## Supplementary Information

Below is the link to the electronic supplementary material.

Supplementary material 1: Vorticity in the axisymmetric case with 60% stenosis and pulsation frequency w3 (MP4 14381 kb)

Supplementary material 2: Vorticity in the axisymmetric case with 60% stenosis and pulsation frequency w2 (MP4 13101 kb)

Supplementary material 3: Vorticity in the axisymmetric case with 60% stenosis and pulsation frequency w1 (MP4 11951 kb)

Supplementary material 4: Vorticity across the x-z plane in the eccentric case with 60% stenosis and pulsation frequency w1 (MP4 9778 kb)

Supplementary material 5: Vorticity across the x-z plane in the eccentric case with 60% stenosis and pulsation frequency w3 (MP4 14491 kb)

Supplementary material 6: Vorticity across the x-z plane in the eccentric case with 60% stenosis and pulsation frequency w2 (MP4 13308 kb)

## Footnotes

1 https://apes.osdn.io.

2 A detailed analysis of Kolmogorov microscales has been omitted because the turbulence intensity in these cases is lower than the previous study (Jain 2020b). Please see the results section for more details.

3 Please refer to the animations in supplementary material for more details.

4 Such observations are more clear in the animations provided as supplementary information.
